# Can a physical activity monitor provide a valid measure of arm elevation angle? A study to assess agreement between the SenseWear Mini Armband and the universal goniometer

**DOI:** 10.1186/s12891-015-0507-4

**Published:** 2015-03-03

**Authors:** Andrew D Hirschhorn, John W Lockhart, John D Breckenridge

**Affiliations:** The Clinical Research Institute, Suite 6, 16 to 18 Mons Road, Westmead, NSW 2145 Australia; Department of Physiotherapy, Westmead Hospital, Sydney, Australia; Department of Physiotherapy, The University of Melbourne, Melbourne, Australia; Physical Therapy Division, University of Kentucky, Lexington, USA; Mungovan Breckenridge Physiotherapy and Associates, Sydney, Australia

**Keywords:** Range of motion, Physical activity monitor, Accelerometer, Upper extremity, Shoulder, Goniometry

## Abstract

**Background:**

We undertook the current study to assess whether an accelerometer-based physical activity monitor, the SenseWear Mini Armband (SMA), could be used to provide data on static arm elevation, and to assess the agreement between static arm elevation measures obtained using SMA-derived data and those obtained with a universal goniometer.

**Methods:**

Using a universal goniometer, healthy adult subjects (n = 25, age 30 ± 9 years) had each of right and left arms positioned in a series of set positions between arm-by-side and maximal active arm flexion (anteversion), and arm-by-side and maximal active arm abduction. Subjects wore the SMA throughout positioning, and SMA accelerometer data was used to retrospectively calculate/derive arm elevation angle using a manufacturer-provided algorithm. The Bland-Altman method was used to assess agreement between goniometer-set and SMA-derived arm elevation angles.

**Results:**

There were significant differences between goniometer-set and SMA-derived arm elevation angles for elevation angles ≤ 30 degrees and ≥ 90 degrees (p < 0.05). Bland-Altman plots showed that the greater the angle of elevation, the greater the mean difference between goniometer-set and SMA-derived elevation angles. Adjustment of the manufacturer-provided algorithm for deriving arm elevation angle corrected for this systematic difference, and resulted in 95% limits of agreement ± 12 degrees (flexion) and ± 13 degrees (abduction) across the full range of arm elevation.

**Conclusions:**

The SMA can be used to record data allowing derivation of static arm elevation angle in the upright position, 95% limits of agreement with the universal goniometer being similar to those reported for digital inclinometers and gyroscopes. Physiotherapists looking for innovative methods of recording upper limb range of motion should consider the potential of accelerometer-based physical activity monitors such as the SMA.

## Background

Physiotherapists routinely measure joint range of motion (ROM) to quantify the flexibility or stiffness of a joint, and as one proxy indicator of musculoskeletal function. Return to ‘normal’ or functional joint ROM is often a key goal of therapeutic intervention [1]. The most common device used by physiotherapists to measure joint ROM in the clinic is the analog, ‘universal’ goniometer [2]. When used across joints with large physiological ROMs, e.g. the glenohumeral joint, the universal goniometer generally demonstrates high intra-rater reliability [3,4]. As such, the universal goniometer has been used as a standard against which to validate/compare alternate measurement devices [1,5,6].

The advent of digital technologies has seen a number of alternate ROM measurement devices become available to physiotherapists. Devices make use of *inter alia* (camera-based) optoelectric systems, digital inclinometers, [5] gyroscopes, [6] accelerometers, [7,8] and/or a combination of such sensors, e.g. in wireless microelectromechanical systems (MEMS) [9]. A common limitation of such devices, and one shared with the universal goniometer, is the need for the physiotherapist or technician to be present at the time of measurement; there is limited scope to measure joint ROM ‘in the field’ , or during non-supervised activity. Measurements obtained in the clinical environment may also be subject to physiotherapist/technician influence or bias, and hence may not truly reflect the effect of pathology on everyday function.

Wearable physical activity monitors are used in healthy and patient populations to assess various aspects of non-supervised activity [10]. Physical activity monitors incorporate accelerometers and data recorders to detect and record device attitude and movement, which are analysed continuously or retrospectively to intuit/calculate e.g. step count and/or metabolic activity [10]. We perceived that such physical activity monitors, should they be affixed to the arm, might be able to calculate elevation of the arm in respect to gravity, and hence provide an indication of upper limb (i.e. combined glenohumeral and scapulothoracic) ROM in the upright position. Benefits would include the ability to record ROM ‘in the field’ , for delayed analysis; and, potentially, the ability to describe ROM limitation over a period of time.

We undertook the current study as a ‘proof of concept’ study, i.e. to assess whether a specific accelerometer-based physical activity monitor, the SenseWear Mini Armband (SMA), could be used to provide data on static arm elevation, and to assess the agreement between static arm elevation measures obtained using the SMA and those obtained with a universal goniometer.

## Methods

### Subjects

Between June and July 2013, healthy adult (> 18 years) subjects were recruited through advertisements placed in the offices of a private-sector physiotherapy practice in Sydney, Australia. Exclusion criteria included current arm, shoulder or hand pathology precluding pain-free active upper limb ROM. Subjects were screened using the QuickDASH, an 11-item questionnaire of arm, shoulder or hand pain/dysfunction, scored between 0 (no pain/dysfunction) and 100 (most severe pain/dysfunction) [11]. If prospective subjects reported a QuickDASH score > 0, the researcher questioned the subject further to ascertain current pain status with unloaded, active upper limb ROM. The study was approved by Western Sydney Local Health District’s Human Research Ethics Committee, and all subjects provided written informed consent before participation.

General demographic data, e.g. date of birth, were obtained directly from subjects. Height and weight were measured using a combined scale/stadiometer (Tanita WB-3000, Wedderburn, Sydney).

### Study protocol

While standing erect, subjects had their arms positioned in a series of 16 positions for each of right then left arms, in the following order:

- Flexion (anteversion): arm-by-side, 30°, 45°, 60°, 90°, 120°, 150°, maximal active ROM
- Abduction: arm-by-side, 30°, 45°, 60°, 90°, 120°, 150°, maximal active ROM.

The scapula was not stabilised, i.e. the set angles of flexion/abduction were achieved using a subject-determined combination of glenohumeral and scapulothoracic motion.

Conventional goniometry (using a 360°, 20 cm clear plastic goniometer) was used to measure flexion/abduction angle in arm-by-side and maximal positions, and to position the arm in the requisite, or ‘set’ angle of flexion/abduction for set positions (“goniometer-set angle”, gΘ). Body landmarks used for goniometry were:

- Flexion: Lateral aspect of acromion and lateral border of the humerus (forearm held in neutral pronation/supination).
- Abduction: Anterior aspect of acromion and midline of humerus (forearm held in neutral pronation/supination).

A plumb line was used to provide a vertical reference line.

Once positioned, subjects maintained each position for 30 s by grasping an adjustable suction-cup/handle affixed to an adjacent wall. The handle was orientated vertically for flexion positions and horizontally for abduction positions to minimise subjects’ muscle activity. Subjects were able to rest between positions as required.

Subjects wore the SMA throughout positioning (on the right arm for right arm flexion/abduction, on the left arm for left arm flexion/abduction). The SMA has a ‘timestamp’ button, which marks its data output electronically, thereby allowing for delineation of data between positions. Once a subject’s arm position had been measured/set, the researcher pressed the timestamp button to commence data collection; 30 s later the researcher again pressed the timestamp button to end data collection.

### The SenseWear Mini Armband

The SMA (BodyMedia Inc., Pittsburgh, USA) is a lightweight (50 g), wireless physical activity monitor worn on the upper arm. The SMA incorporates a manufacturer-calibrated, tri-axial accelerometer and physiological sensors, data from which are used to calculate step count and energy expenditure according to proprietary ‘black box’ algorithms. Data from the tri-axial accelerometer can be retrospectively downloaded and obtained independently of physiological data, using SenseWear Professional Software (version 7.0). Raw accelerometer data are initially obtained in the form ‘x, y, z’ , where −1 ≤ x, y, z ≤ 1, representing acceleration of the SMA in transverse (pitch), forward (roll), and longitudinal axes, relative to gravity.

**Figure 1:**
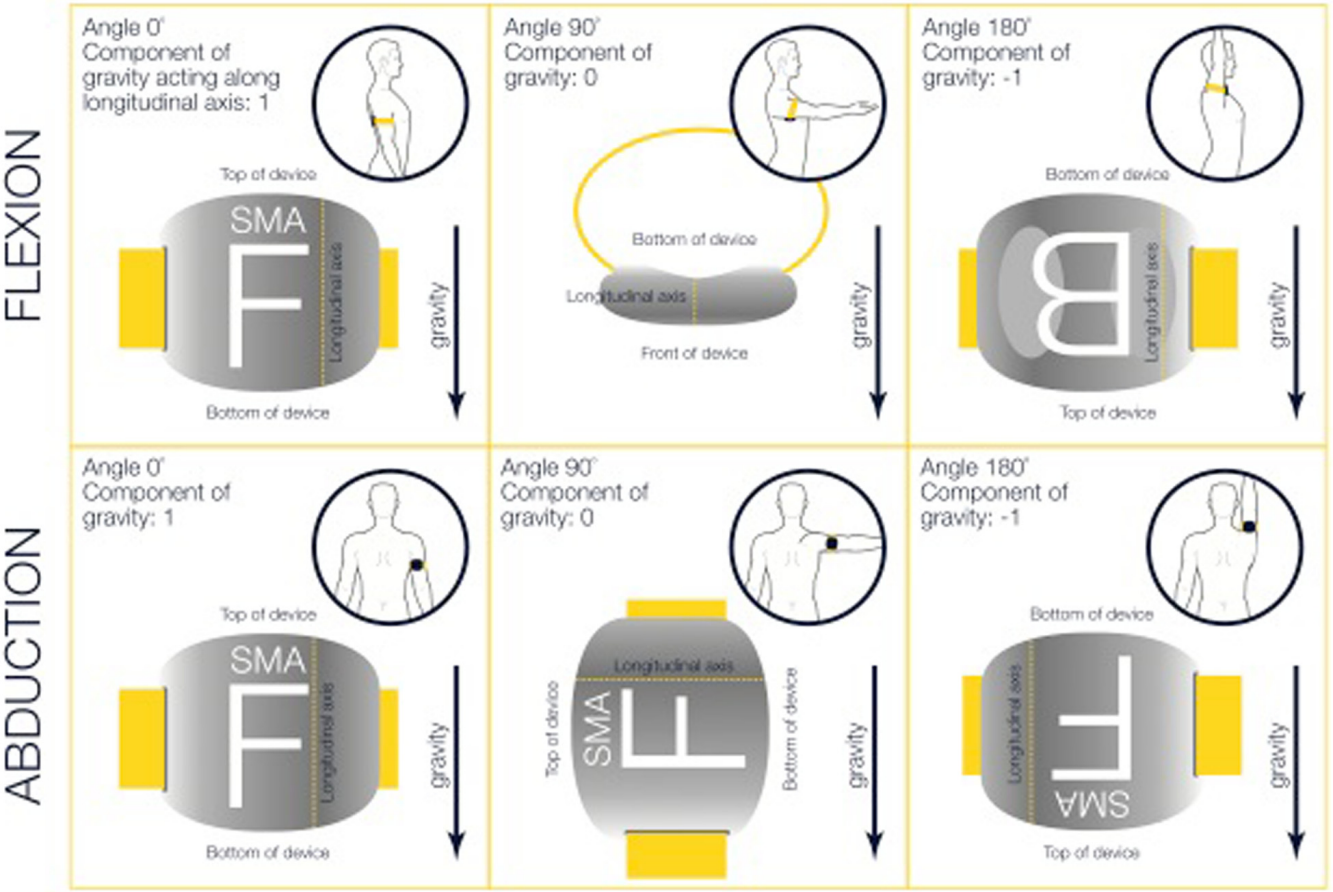
**Theoretical longitudinal accelerometer data associated with displacement of the SMA with respect to gravity.** The longitudinal axis of the SMA is the theoretical line joining the top and bottom of the device. F: front (outer face) of device; B: back (inner face) of device.

When the SMA is stationary (e.g. is in a set position), accelerometer data represent the component of gravity acting on the SMA along each axis. Hence when the SMA is stationary and vertically upright, the longitudinal accelerometer (z) should theoretically read at 1 (the full component of gravity acting along the longitudinal axis), when stationary and horizontal, 0 (gravity acting perpendicular to the longitudinal axis), and when stationary and vertically upside down, −1 (the full component of gravity again acting along the longitudinal axis (see Figure 1). The manufacturer-provided algorithm for determining angular displacement of the SMA relative to gravity, i.e. from the vertical plane (elevation), is: elevation (“SMA-derived elevation angle”, a_1_Θ) = cos^−1^(z) [12].

In this study we obtained longitudinal accelerometer data (z) at a frequency of 1 Hz (i.e. we obtained 30 data points for each position for each subject). The 30 data points for each position were averaged to provide a single time-averaged data point. The time-averaged z data points were then transformed to angular elevation angle (a_1_Θ) using the manufacturer-provided algorithm.

The position of the SMA on the upper arm was standardised as follows: the subject was asked to perform resisted shoulder abduction in the functional position to allow for palpation of the deltoid tuberosity; marks were then placed on the subject’s arm at the location of the deltoid tuberosity and on the posterior aspect of the arm directly in the line of the deltoid tuberosity. The SMA was then centred vertically over the posterior marker using the manufacturer-supplied velcro armband.

### Statistical analysis

The statistics package IBM SPSS Statistics Version 20 was used to analyse data. Two–tailed tests with a 5% significance level were used throughout. Simple descriptive statistics were used to summarise subject data and SMA-derived elevation angle (a_1_Θ) for goniometer-set shoulder positions. Differences between goniometer-set (gΘ) and SMA-derived elevation angle (a_1_Θ) were calculated for each of flexion and abduction, and ANOVAs performed to investigate if differences were associated with subject and study condition (left vs right arm, flexion vs abduction).

Bland-Altman plots were used to assess agreement between gΘ and a_1_Θ for each of arm flexion and abduction [13]. The Pearson product–moment correlation statistic was used to measure correlations between gΘ and a_1_Θ.

*A priori* sample size calculations suggested that 200 data points for each study condition, i.e. 25 subjects by 8 positions in each of left arm flexion, left arm abduction, right arm flexion and right arm abduction, would allow us to calculate confidence intervals ± 0.24 sd for the 95% limits of agreement between gΘ and a_1_Θ (where sd is the standard deviation of the differences between gΘ and a_1_Θ).

## Results

### Demographics

25 subjects (11 male, 14 female, age: 30 ± 9 years, body mass index: 24.0 ± 3.2 kg.m^−2^) participated in the study. Twenty-two subjects (88%) recorded a QuickDASH score of 0; three subjects (12%) recorded a QuickDASH score > 0. Further questioning of these three subjects revealed mild pain/symptoms and/or slight difficulty with functional activities related to a minor hand injury (n = 1), previous shoulder injury (n = 1), and a history of anterior cervical discectomy/fusion (n = 1). None reported pain with, or limitation of, unloaded active upper limb ROM. Data for all subjects were included in the current analysis.

One subject was unable to achieve > 150 degrees of right arm abduction, left arm flexion and left arm abduction (as measured with the goniometer), therefore ‘maximal’ active ROM measures for those movements were not recorded.

### Agreement between goniometer-set and SenseWear Armband-derived arm elevation angles

Mean values for gΘ and a_1_Θ for each study condition are presented in Table 1. There was no significant effect of subject on differences between gΘ and a_1_Θ for flexion (p = 0.129), but there was a significant effect of subject on differences for abduction (p = 0.002). There was no significant effect of condition (left vs right, flexion vs abduction) on differences between gΘ and a_1_Θ (p = 0.173).

**Table 1:** **Values for goniometer-set and SMA-derived arm elevation angles (mean ± sd)**

**Goniometer-set angle (gΘ) (degrees)**	**SMA-derived angle (a**_**1**_ **Θ) (degrees)**		**Goniometer-set angle (gΘ) (degrees)**	**SMA-derived angle (a**_**1**_ **Θ) (degrees)**	
	Right arm flexion	Left arm flexion		Right arm abduction	Left arm abduction
Arm-by-side* (right: 1 ± 1, left: 2 ± 2)	14 ± 3^a^	15 ± 5^a^	Arm-by-side* (right: 7 ± 2, left: 6 ± 2)	13 ± 3^a^	14 ± 5^a^
30	33 ± 5^b^	33 ± 5^b^	30	27 ± 6^b^	31 ± 5
45	46 ± 4	45 ± 5	45	44 ± 5	46 ± 5
60	59 ± 4	59 ± 6	60	59 ± 6	59 ± 6
90	83 ± 5^a^	80 ± 6^a^	90	83 ± 5^a^	83 ± 5^a^
120	110 ± 4^a^	108 ± 5^a^	120	111 ± 4^a^	108 ± 5^a^
150	134 ± 5^a^	132 ± 6^a^	150	135 ± 4^a^	130 ± 4^a^
Maximal active ROM* (right: 164 ± 5, left: 165 ± 4)	147 ± 5^a^	145 ± 6^a^	Maximal active ROM* (right: 169 ± 4, left: 170 ± 4)	a_1_Θ = 149 ± 5^a^	a_1_Θ = 145 ± 6^a^

ROM: range of motion.

*Note that there was variation between individuals, and for individuals between left and right shoulders, for gΘ for ‘arm-by-side’ and ‘maximal active ROM’ positions.

^a^p < 0.001 vs gΘ; ^b^p < 0.05 vs gΘ.

**Figure 2:**
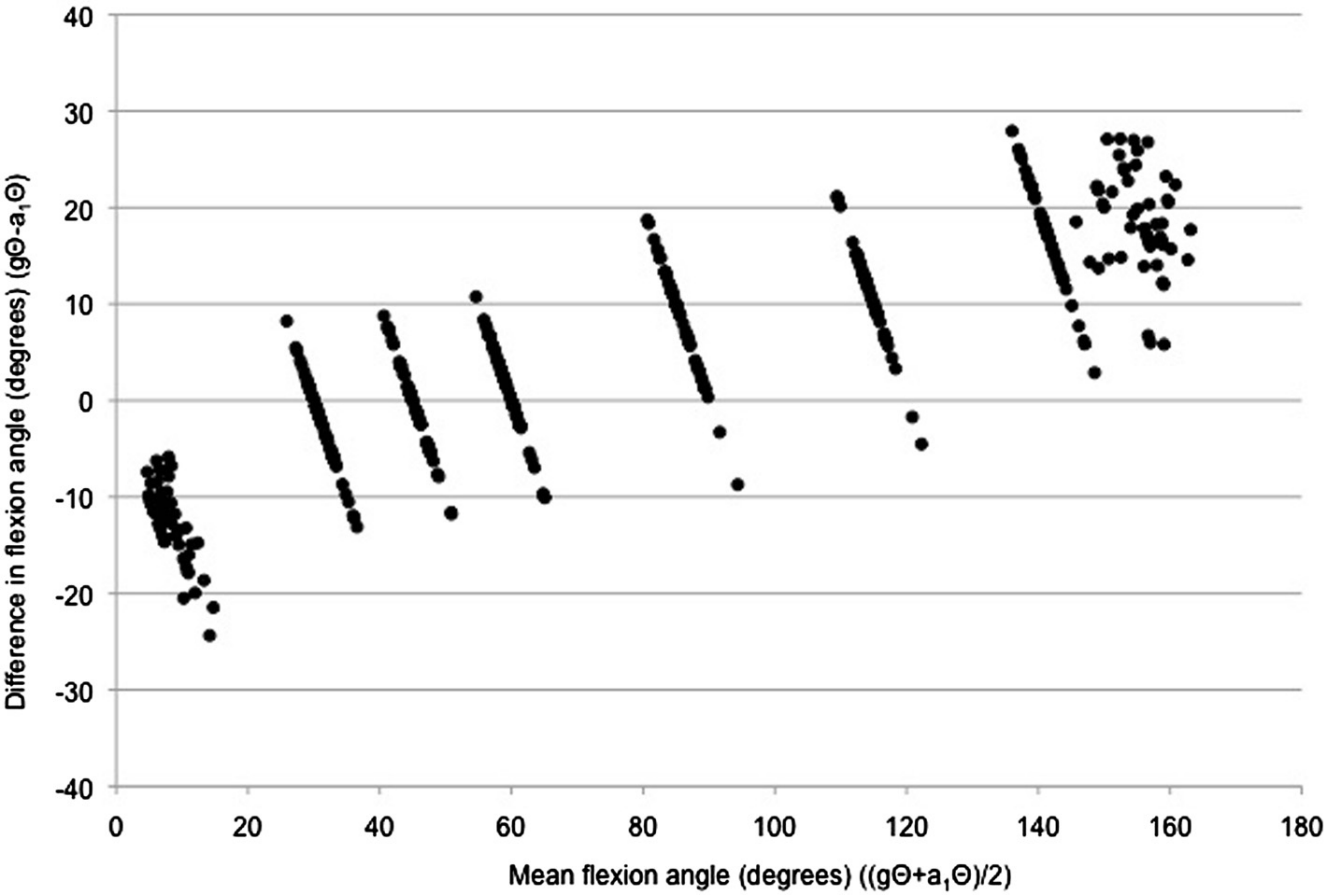
**A Bland-Altman plot of the difference between goniometer-set elevation angle (gΘ) and SMA-derived elevation angle (a**_**1**_ **Θ) plotted against the mean of gΘ and a**_**1**_ **Θ, for flexion data points.**

**Figure 3:**
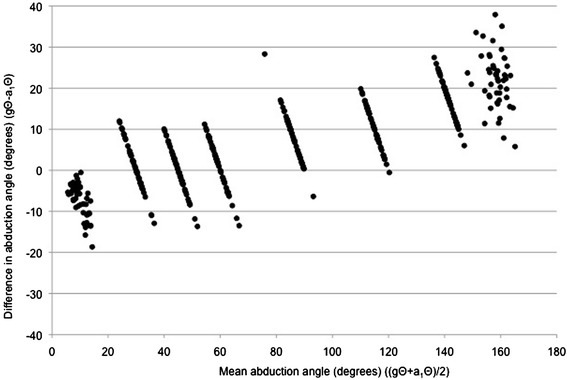
**A Bland-Altman plot of the difference between goniometer-set elevation angle (gΘ) and SMA-derived elevation angle (a**_**1**_ **Θ) plotted against the mean of gΘ and a**_**1**_ **Θ, for abduction data points.**

Figures 2 and 3 are Bland-Altman plots of the difference between gΘ and a_1_Θ plotted against the mean of gΘ and a_1_Θ for arm flexion and arm abduction data points respectively. Visual inspection of the Bland-Altman plots revealed obvious relationships between the difference and the mean, i.e. the greater the mean elevation angle (ergo in this study also the goniometer-set elevation angle), the greater the discrepancy between goniometer-set and SMA-derived angles (flexion: r^2^ = 0.742, p < 0.001; abduction: r^2^ = 0.665, p < 0.001).

**Figure 4:**
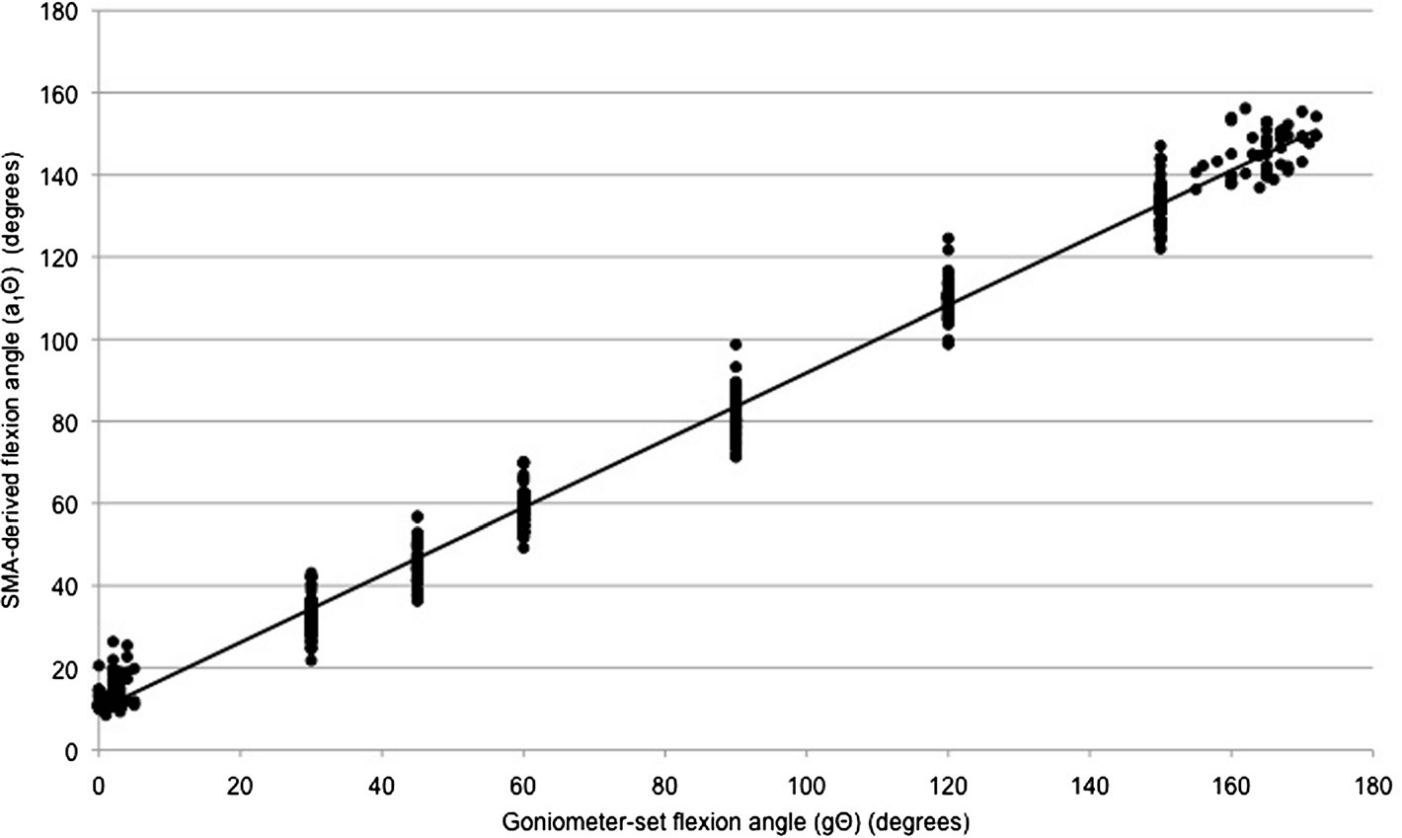
**A scatter-plot of goniometer-set elevation angle (gΘ) vs SMA-derived elevation angle (a**_**1**_ **Θ), for flexion data points.**

**Figure 5:**
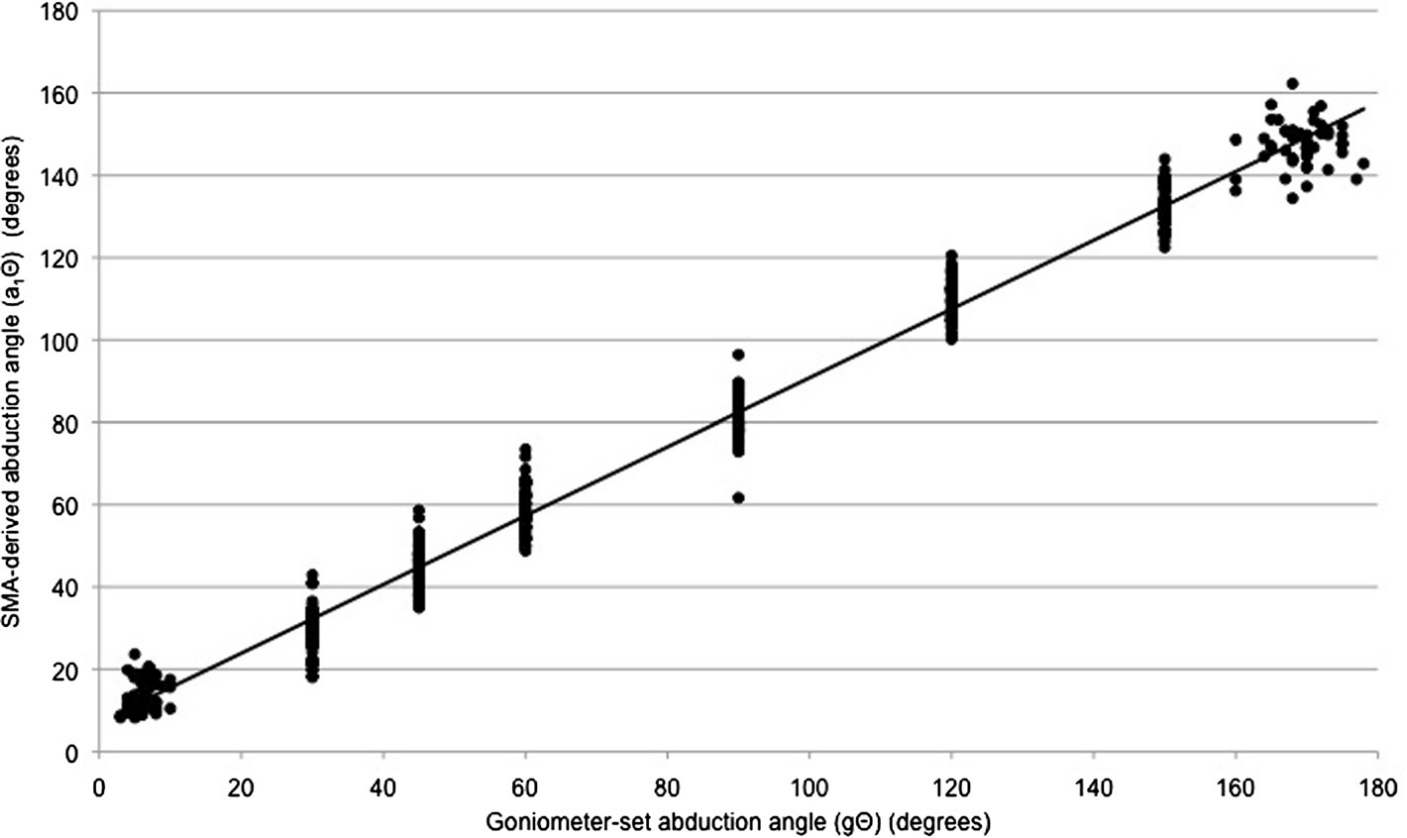
**A scatter-plot of goniometer-set elevation angle (gΘ) vs SMA-derived elevation angle (a**_**1**_ **Θ), for abduction data points.**

Figures 4 and 5 are scatter-plots of gΘ vs a_1_Θ for arm flexion and arm abduction data points respectively. There were significant and very strong correlations between gΘ and a_1_Θ (flexion: r^2^ = 0.987, p < 0.001; abduction: r^2^ = 0.985, p < 0.001). The equations for the lines of best fit were: flexion: a_1_Θ = 0.821 * (gΘ) + 9.7373; abduction: a_1_Θ = 0.8367 * (gΘ) + 7.1269.

These equations were subsequently applied to SMA-derived angles (a_1_Θ) to calculate “adjusted” SMA-derived angles (a_2_Θ), *viz*: flexion: a_2_Θ = (a_1_Θ – 9.7373)/0.821; abduction: a_2_Θ = (a_1_Θ – 7.1269)/0.8367.

**Figure 6:**
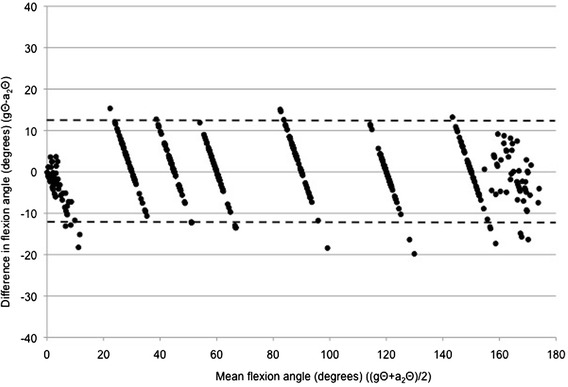
**A Bland-Altman plot of the difference between goniometer-set elevation angle (gΘ) and the adjusted SMA-derived elevation angle (a**_**2**_ **Θ) plotted against the mean of gΘ and a**_**2**_ **Θ, for flexion data points.**

**Figure 7:**
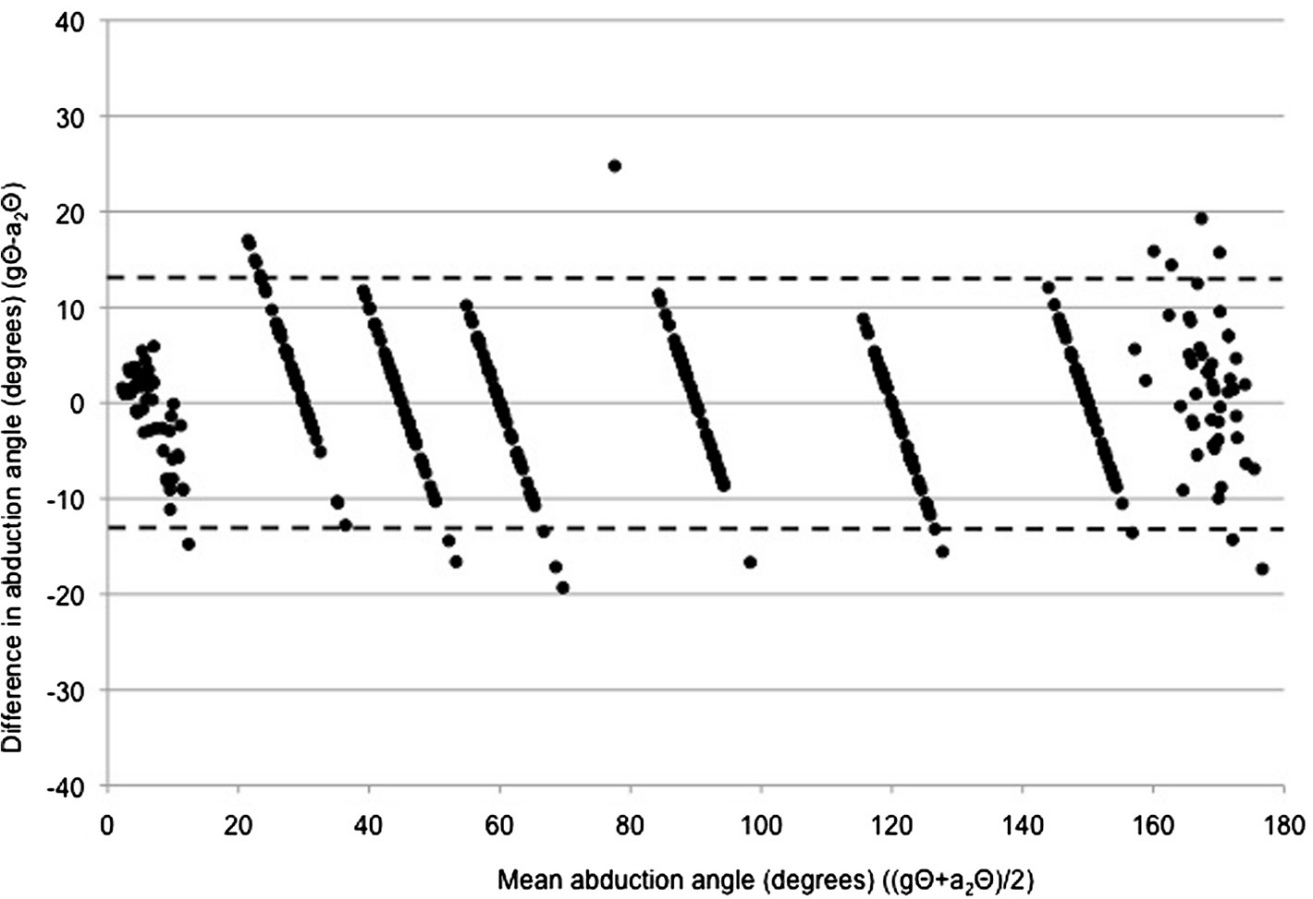
**A Bland-Altman plot of the difference between goniometer-set elevation angle (gΘ) and the adjusted SMA-derived elevation angle (a**_**2**_ **Θ) plotted against the mean of gΘ and a**_**2**_ **Θ, for abduction data points.**

Figures 6 and 7 are repeat Bland-Altman plots of the difference between gΘ and the adjusted SMA-derived angle (a_2_Θ) plotted against the mean of gΘ and a_2_Θ, for flexion and abduction data points respectively. Visual inspection of the Bland-Altman plots showed corrections of the previously observed systematic relationships between the difference and the mean.

The mean difference between gΘ and a_2_Θ was 0 degrees for both flexion and abduction, because of the correction constants in the algorithms used to calculate a_2_Θ. The standard deviations of the observed differences (flexion: 6.22 degrees; abduction: 6.71 degrees) were used to calculate 95% confidence intervals for the mean difference (flexion: ± 12 degrees; abduction: ± 13 degrees), illustrated on the Bland-Altman plots with dotted lines.

## Discussion

The primary aims of the current study were: i) to demonstrate proof of concept, that an accelerometer-based physical activity monitor (the SMA) could be used to provide retrospective data on static arm elevation angle, hence an indication of upper limb ROM; and ii) to assess agreement between static arm elevation measures obtained using the SMA and those obtained with a universal goniometer. We found that we could derive static arm elevation measures from SMA data, and indeed there were significant and very strong correlations between measures derived using SMA data and those set with the universal goniometer. The use of a manufacturer-provided algorithm to calculate arm elevation from SMA data, however, resulted in a progressive discrepancy between the two methods of measurement, i.e. the greater the goniometer-set elevation angle, the greater the mean difference between goniometer-set and SMA-derived angles. This progressive discrepancy could be corrected for by adjusting the algorithm used to derive angles from SMA data, resulting in limits of agreement ± 12 degrees (flexion) and ± 13 degrees (abduction) across the full range of arm elevation.

A likely explanation for the progressive discrepancy observed with increasing arm elevation in the current study relates to progressive ‘tilting’ of the SMA relative to gravity and the virtual line joining bony landmarks. It has been observed that both arm flexion, and to a greater degree, arm abduction are associated with adjunct humeral rotation [14]. While not measured in the current study, said humeral rotation might be expected to progressively alter SMA attitude, notwithstanding that surface tissue may not rotate precisely in concert with underlying bony structures.

Similarly, uneven ‘seating’ of the SMA on the contours of each subject’s upper arm may have contributed to SMA tilt, hence to the observed progressive discrepancy. The upper arm only approximates a cylindrical shape; subcutaneous muscle distorts the skin surface. Our experimental set-up was designed to minimise muscle contraction in each static position, but passive lengthening and shortening of opposing upper arm muscle groups as the arm was repositioned in progressively more elevated positions might also have affected SMA attitude. That the relationship between goniometer-set and SMA-derived angles was subject-dependent (for abduction) supports this ‘uneven seating’ hypothesis, as individual subjects differed in their upper arm morphology. Alternative but unlikely explanations for the progressive discrepancy include that the researcher setting arm elevation with the universal goniometer systematically worsened in accuracy as the elevation angle increased, or that the SMA’s accelerometer itself introduced a systematic error as it rotated around its longitudinal axis.

Several previous studies have reported on the agreement between other proprietary accelerometer-based devices and criterion standard devices for the measurement of arm and/or shoulder elevation. Bernmark & Wiktorin measured agreement between a tri-axial accelerometer device (Logger Technology HB) and an optoelectric measuring system across a range of shoulder flexion and abduction positions (0 to 180 degrees), [7] finding ‘almost perfect correspondence’ between measures taken with the two devices. Notably, the accelerometer device was calibrated for each subject prior to testing. Amasay et al. validated a similar tri-axial accelerometer device (the Virtual Corset) against a digital protractor, [15] seating both devices on a vice rather than on a human arm; for measurement of static angles the Virtual Corset differed by up to 14 degrees from the digital protractor, the largest differences occurring at 0 and 180 degrees of elevation.

Kolber et al. and El-Zayat et al. reported on agreement between other, non-accelerometer based digital devices (a digital inclinometer and a three-dimensional gyroscope respectively) and the universal goniometer, for the measurement of end shoulder flexion and abduction ROM in healthy subjects [5,6]. Both studies reported ‘acceptable’ agreement with the universal goniometer (95% limits of agreement: flexion: −20 to 5 degrees, −8 to 9 degrees; abduction: −17 to 14 degrees). The ROM over which agreement was measured in both studies was limited (Kolber et al. [5]: goniometer-measured flexion: mean 156 ± 9 degrees; abduction mean 161 ± 11 degrees; El-Zayat et al. [6]: goniometer-measured flexion: range: 129 to 186 degrees; abduction: 116 to 186 degrees).

A comparative strength of the current study is that we assessed agreement between the SMA and the universal goniometer across the full range of arm elevation angles (arm-by-side to maximal active elevation). We note that in practice, physiotherapists typically *are* interested in maximal ROM, as an objective descriptor of functional limitation. In the presence of pathology however, arm elevation is often limited, certainly to less than that tested in previous studies. Given the progressive discrepancies observed in the current study, agreement between devices for maximal ROM measurement in healthy subjects might not assure agreement for maximal ROM measurement in patients with pathology. By way of example, if we had only examined data at goniometer-set elevation angles of 45 and 60 degrees in the current study (see Table 1), we would have reported an unjustifiably high level of agreement between the universal goniometer and the SMA.

Are the limits of agreement observed in the current study ‘tight’ enough to commend the use of the SMA as a valid alternative to universal goniometry in the clinical setting? Arguably not, if one considers the universal goniometer itself to be a valid criterion standard measure of arm elevation. A potential variation of 24 to 26 degrees (± 12 to 13 degrees) around the ‘true’ value of arm elevation is similar to that observed for the digital inclinometer and gyroscope, but would likely be perceived by physiotherapists as being unsatisfactory if one considered a 5 degree change in ROM to be clinically significant [1]. As noted by Kolber et al. however, there are limited data on the validity of the universal goniometer against e.g. radiographic measures of movement, [5] – the argument could be drawn that digital devices should rather be considered the criterion standard against which goniometry should be compared.

One clinical benefit of using a physical activity monitor with data recorder, such as the SMA, is that a physiotherapist could measure e.g. daily progression of ROM limitation, without the need for a patient to physically attend the clinic. Patients could flex/abduct the arm to its limit at pre-defined intervals, ‘mark’ SMA data electronically using the timestamp button, and return the SMA to the clinic *ex post facto* for data download and analysis. An important consideration in such use would be the patient’s capacity to ensure consistent placement of the SMA on the arm. If one could account for dynamic acceleration of the arm, as discussed by Amasay et al. [15] theoretically one could also retrospectively track arm elevation over a period of time, e.g. to assess work-related arm movement demands or patient adherence to physiotherapist-imposed arm movement restrictions. Such clinical benefits might offset the relatively high cost of the SMA (AU$1722 including software at the time of study conduct) compared to the universal goniometer.

A weakness of the current study is that we calculated adjusted SMA-derived arm elevation angles using the same algorithms for all subjects. As described, however, there was a significant effect of subject on differences between the goniometer-set and SMA-derived elevation angles for abduction. Calibration of the SMA for individual patients (i.e. use of patient-specific algorithms) might provide for better agreement with the universal goniometer in the clinical/field setting - albeit if a patient did have limited range, said calibration would be problematic.

A second weakness of the current study is that we did not assess the reliability of arm elevation measures derived from the SMA. As an electronic device, we would argue that any unreliability we might have observed would only be related to the reliability of the researcher/universal goniometer in setting elevation angles (neither did we assess inter-tester reliability of this), and/or alterations in how the SMA was seated on the arm. Nonetheless, further research into the clinical feasibility of the SMA to measure/record arm elevation should include assessment of both inter- and intra-tester reliability.

## Conclusions

We have demonstrated that a commercially available physical activity monitor, the SMA, can be used to record and provide data on static arm elevation in the upright position. There was a significant and very strong correlation between arm elevation angles as derived from SMA data and set with a universal goniometer. A progressive discrepancy between measures obtained using the two devices/methods could be corrected for, resulting in agreement between the two devices across a full ROM similar to that reported for digital inclinometers and gyroscopes. Physiotherapists looking for innovative methods of recording patient upper limb ROM outside of the clinic should consider the potential of wearable, accelerometer-based physical activity monitors.

## Abbreviations

a_1_Θ: SMA derived elevation angle
a_2_Θ: Adjusted SMA derived elevation angle
gΘ: Goniometer-set angle
B: Back (inner face) of SenseWear Mini Armband
F: Front (outer face) of SenseWear Mini Armband
MEMS: Microlectromechanical systems
ROM: Range of motion
SMA: SenseWear Mini Armband
